# Quantitative Modelling of Trace Elements in Hard Coal

**DOI:** 10.1371/journal.pone.0159265

**Published:** 2016-07-20

**Authors:** Adam Smoliński, Natalia Howaniec

**Affiliations:** Department of Energy Saving and Air Protection, Central Mining Institute, Katowice, Poland; IUMPA—Universitat Politecnica de Valencia, SPAIN

## Abstract

The significance of coal in the world economy remains unquestionable for decades. It is also expected to be the dominant fossil fuel in the foreseeable future. The increased awareness of sustainable development reflected in the relevant regulations implies, however, the need for the development and implementation of clean coal technologies on the one hand, and adequate analytical tools on the other. The paper presents the application of the quantitative Partial Least Squares method in modeling the concentrations of trace elements (As, Ba, Cd, Co, Cr, Cu, Mn, Ni, Pb, Rb, Sr, V and Zn) in hard coal based on the physical and chemical parameters of coal, and coal ash components. The study was focused on trace elements potentially hazardous to the environment when emitted from coal processing systems. The studied data included 24 parameters determined for 132 coal samples provided by 17 coal mines of the Upper Silesian Coal Basin, Poland. Since the data set contained outliers, the construction of robust Partial Least Squares models for contaminated data set and the correct identification of outlying objects based on the robust scales were required. These enabled the development of the correct Partial Least Squares models, characterized by good fit and prediction abilities. The root mean square error was below 10% for all except for one the final Partial Least Squares models constructed, and the prediction error (root mean square error of cross–validation) exceeded 10% only for three models constructed. The study is of both cognitive and applicative importance. It presents the unique application of the chemometric methods of data exploration in modeling the content of trace elements in coal. In this way it contributes to the development of useful tools of coal quality assessment.

## Introduction

In the world of growing energy demand and increasing awareness of sustainable development coal still remains the dominant fossil fuel representing the significant share in energy supply [1]. The environmental concerns and economic factors related to the present-day coal mining and energy sectors are the main driving forces of the development of solutions contributing to the more sustainable management of natural resources, as well as cleaner and more efficient energy generation and utilization. Here the research field related to the highly efficient thermochemical conversion of solid fuels, often in co-processing with bio- and industrial waste prevails [2,3], with the special focus on hydrogen production as a prospective energy carrier [4–6]. An attention is also given to the potential recycling of valuable elements from coal ash [7,8]. Inevitably, this vast amount of research and applications need to be supported by the development of suitable analytical solutions, including efficient methods of experimental, operating and monitoring data sets exploration and modeling.

In the paper the application of the advanced chemometric methods in the modeling of coal quality parameters is presented. The robust Partial Least Squares method has been proved to be effective in modeling the relationships between the contents of trace elements and the remaining physical and chemical parameters of coal and coal ash.

In the previous study [9] the analysis of dissimilarities between coal samples of various zones of the Upper Silesian Coal Basin (USCB) with the application of the Hierarchical Clustering Analysis was performed. The data set explored included basic physical and chemical parameters, including trace elements contents and the geological factors applicable for particular zones of coal seams location. In the present study the quantitative relationships between concentration of trace elements (As, Ba, Cd, Co, Cr, Cu, Mn, Ni, Pb, Rb, Sr, V and Zn) in 132 coal samples provided by 17 coal mines of the USCB, the remaining physical and chemical parameters of coal and coal ash components were analyzed with the application of the Partial Least Squares method. The model developed is characterized by good fit and prediction abilities and may be considered as a useful tool of coal quality assessment, in particularly in terms of the environmental aspect of coal utilization in thermochemical processing. The study is of both cognitive and applicative importance. First, it provides the direct information on characteristics of a wide range of bituminous coals of the largest coal basin in Poland. More importantly however, it presents the unique application of the chemometric methods of data exploration in modeling the content of trace elements in coal, based on the basic physical and chemical characteristics of a fuel.

## Methods

The calibration models are developed principally to describe the relationships between the experimental data, and more importantly, to correctly predict the values of the parameters modeled for the new objects. The most commonly applied linear calibration models for multivariate data include Multivariate Linear Regression (MLR) [10], Principal Components Regression Method (PCR) [10,11], Partial Least Squares Method (PLS) [10,12–18], and Uninformative Variable Elimination–Partial Least Squares method (UVE-PLS) [19,20].

The PLS method is applied in construction of calibration models for multivariate data set, of potentially correlated parameters. New variables, so called latent variables, are constructed here in the way to maximize the description of the covariance between **X** and **y**. The detailed steps of the PLS algorithm are given elsewhere [10].

The model constructed is assessed in terms of how well the predicted value of the dependent variable fits the relevant experimental value for objects from the model set, as well as in terms of predictive abilities for the new data. The Root Mean Square Error (RMS) is calculated to evaluate the fit ability:

$$\text{RMS}=\sqrt{\frac{\sum_{\text{i}=1}^{\text{m}}{\left({\text{y}}_{\text{i}}-{\hat{\text{y}}}_{\text{i}}\right)}^{2}}{\text{m}}}$$ (1)

where y_i_ and ${\hat{\text{y}}}_{\text{i}}$ are the experimental values of the dependent variable for the model set and their predicted values, respectively, while m is the number of objects in the model set.

The predictive ability of a PLS model is assessed based on the Cross Validation (CV) and the Root Mean Square Error of Cross − Validation (RMSCV):

$$\text{RMSCV}\;(\text{A})=\sqrt{\frac{\sum_{\text{i}=1}^{\text{m}}{\left({\text{y}}_{\text{i}}^{\text{t}}-\overset{}{{\hat{\text{y}}}_{\text{i}}^{\text{t}}}\left(\text{A}\right)\right)}^{2}}{\text{m}}}$$ (2)

where ${\text{y}}_{\text{i}}^{\text{t}}$ and ${\hat{\text{y}}}_{\text{i}}^{\text{t}}(\text{A})$ mean respectively: the values from the test set and the respective values predicted by the model of A complexity. Based on the values of the RMSCV not only the assessment of the predictive ability of the model may be performed but also the determination of the optimal number of components required for the model construction. Namely, the number of the components for which the minimum RMSCV value is observed is the optimal one.

The PLS model, similarly to the remaining calibration models, is sensitive to the presence of outliers or outlying objects in the data set, which may result in the weak relationships between the parameters measured. Among the outliers, the ones called leverage points along the x or y directions could be distinguished [21]. They may include “good” or “bad” outliers. The “good” ones are considered to be the outliers that despite the fact they are located far from the majority of the objects, they are still well represented by the model describing the remaining objects. In contrast, the “bad” outliers destabilize the structure of the model constructed. The presence of outliers in the data set implies the need for the application of the robust multiple regression methods, such as the Least Mean Squares (LMS) [21–24], the Repeated Median (RM) [25], the Least Trimmed Squares (LTS) [26], the robust Principal Component Regression (rPCR) [27] and the robust Partial Least Squares (rPLS) methods [28–34].

The rPLS [28–34] enables the construction of the model describing the majority of the good data. To achieve that, a subset of objects free of outliers and including at least 51% of the objects analyzed, is selected. Next, the model of good fit and predictive abilities is constructed for such a “clean subset”. The robust models are constructed based on the Evolutionary Programming (EP) [35], which is the variation of the Genetic Algorithm (GA) [36,37]. Both, the GA and EP, represent the global optimization methods and are based on the principles of the evolution theory, i.e. natural selection and heredity mechanisms.

The EP algorithm in case of rPLS is applied in the selection of a “clean subset” of objects.

The outliers are determined based on the robust scale proposed by Rousseeuw and Leroy [21]. The estimator e_0_ is applicable, defined as:

$${\text{e}}_{0}=1.4826\left(1+\frac{5}{\text{m - g}}\right){\left(\text{median}\left(r_{}^{2}\right)\right)}^{\frac{1}{2}}$$ (3)

where m is the number of objects, g is the number of estimators (e.g. for line g = 2, for plane g = 3), and **r** is the residual vector from the constructed rPLS model.

The i-th object is considered to be the outlier provided that:

$$\frac{{|\text{r}}_{\text{i}}|}{{\text{e}}_{0}}>2.5\;\text{for}\;\text{i}=1,\ldots ,\text{m}$$ (4)

The object identified as an outlier is removed from the data set and the procedure is repeated for the remaining m’ objects. The further estimators (e*) are calculated as:

e*=(∑iri2m'-g)12fori=1,…,m’ (5)

where m’ determines the number of objects after the elimination of the objects identified as the outlier(s) in the preceding step. Next, the data set is checked against any remaining outliers:

$$\frac{{|\text{r}}_{\text{i}}|}{{\text{e}}^{*}}>2.5$$ (6)

### Data

**Fig 1:**
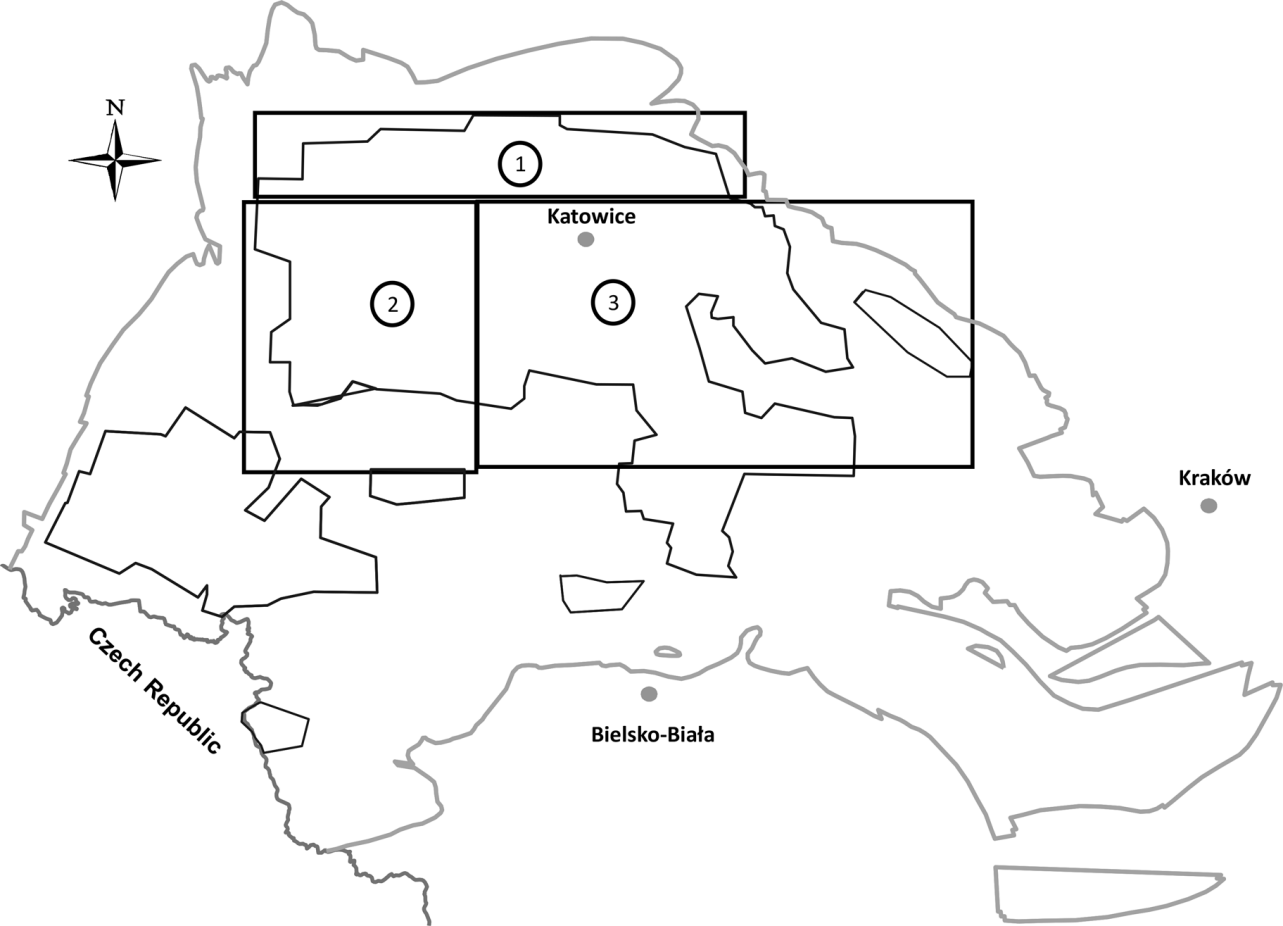
A map of locations of coal sampling sites. Division of the explored area: 1—north zone (objects nos 1–34), 2—west zone (objects nos 35–56), 3 –central zone (objects nos 57–132).

The studied hard coal samples were provided by 17 coal mines located in various zones of the USCB, the largest coal basin in Poland covering the area of 5,600 km^2^ and over 80% of the domestic coal reserves (see Fig 1). The productive carbon overburden of the USCB constitute in main insulating Miocene and Pliocene formations of the thickness of up to 1,100 m. The lower, Paralic series of productive Carboniferous formations are located on marine sediments, and the upper, continental deposits (sandstone and mudstone series) on paralic hiatal surface. The base of the USCB is composed of Precambrian, Cambrian, Devonian and younger Carboniferous rocks [38]. More detailed characterization of the geological settings of the USCB is given in [9].

The data was organized in matrices **X**(132 × 24) and **Y**(132 × 13). The rows of matrices **X** and **Y** (called further objects) represent coals samples of: coal mine 1 (objects nos. 1 and 2), coal mine 2 (objects nos. 3–6), coal mine 3 (objects nos. 7–13), coal mine 4 (objects nos. 14–18), coal mine 5 (objects nos. 19–24), coal mine 6 (objects nos. 25–34), coal mine 7 (objects nos. 35–37), coal mine 8 (objects nos. 38–41), coal mine 9 (objects nos. 42–44), coal mine 10 (objects nos. 45–56), coal mine 11 (objects nos. 57–63), coal mine 12 (objects nos. 64–73), coal mine 13 (objects nos. 74–96), coal mine 14 (objects nos. 97–103), coal mine 15 (objects nos. 104–113), coal mine 16 (objects nos. 114–119), and coal mine 17 (objects nos. 120–132). The columns in matrix **X** represent the parameters describing the physical and chemical parameters of coal and coal ash components listed in Table 1, whereas the columns in matrix **Y** represent the concentrations (μg/g) of trace elements: As, Ba, Cd, Co, Cr, Cu, Mn, Ni, Pb, Rb, Sr, V and Zn (parameters nos. 1–13) in coal. The values of the parameters were determined in the accredited laboratories of the Central Mining Institute, in accordance with the relevant standards regarding solid fuels characteristics: PN-G-04560:1998 (total moisture, ash), PN-G-04516:1998 (volatiles), PN-ISO 1928:2002, based on ISO 1928:1995 (heat of combustion, calorific value), PN-G-04584:2001 (sulfur), PN-G-04571:1998 (carbon), PN-ISO 11724:2008, based on ISO 11724:2004 (fluorine), and PN-G-04534:1999 (chlorine) (see S1 Table). The contents of trace elements were determined with the application of the Inductively Coupled Plasma–Atomic Emission Spectrometry (ICP-OES) and coal ash components with the use of the Wavelength Dispersive X-ray Fluorescence Spectrometry (see S2 Table).

**Table 1:** Physical and chemical parameters of coals and coal ash components included in matrix X(132 x 24).

No.	Parameter	Unit
1	Moisture W	%w/w
2	Ash A	%w/w
3	Volatiles V	%w/w
4	Total sulfur S_t_	%w/w
5	Heat of combustion Q_s_	J/g
6	Calorific value Q_i_	J/g
7	Carbon C_t_	%w/w
8	Pyritic sulfur, S_p_	%w/w
9	SiO_2_	%w/w
10	Al_2_O_3_	%w/w
11	Fe_2_O_3_	%w/w
12	CaO	%w/w
13	MgO	%w/w
14	Na_2_O	%w/w
15	K_2_O	%w/w
16	SO_3_	%w/w
17	TiO_2_	%w/w
18	P_2_O_5_	%w/w
19	BaO	%w/w
20	Mn_3_O_4_	%w/w
21	SrO	%w/w
22	ZnO	%w/w
23	Cl	%w/w
24	F	%w/w

## Results and Discussion

**Fig 2:**
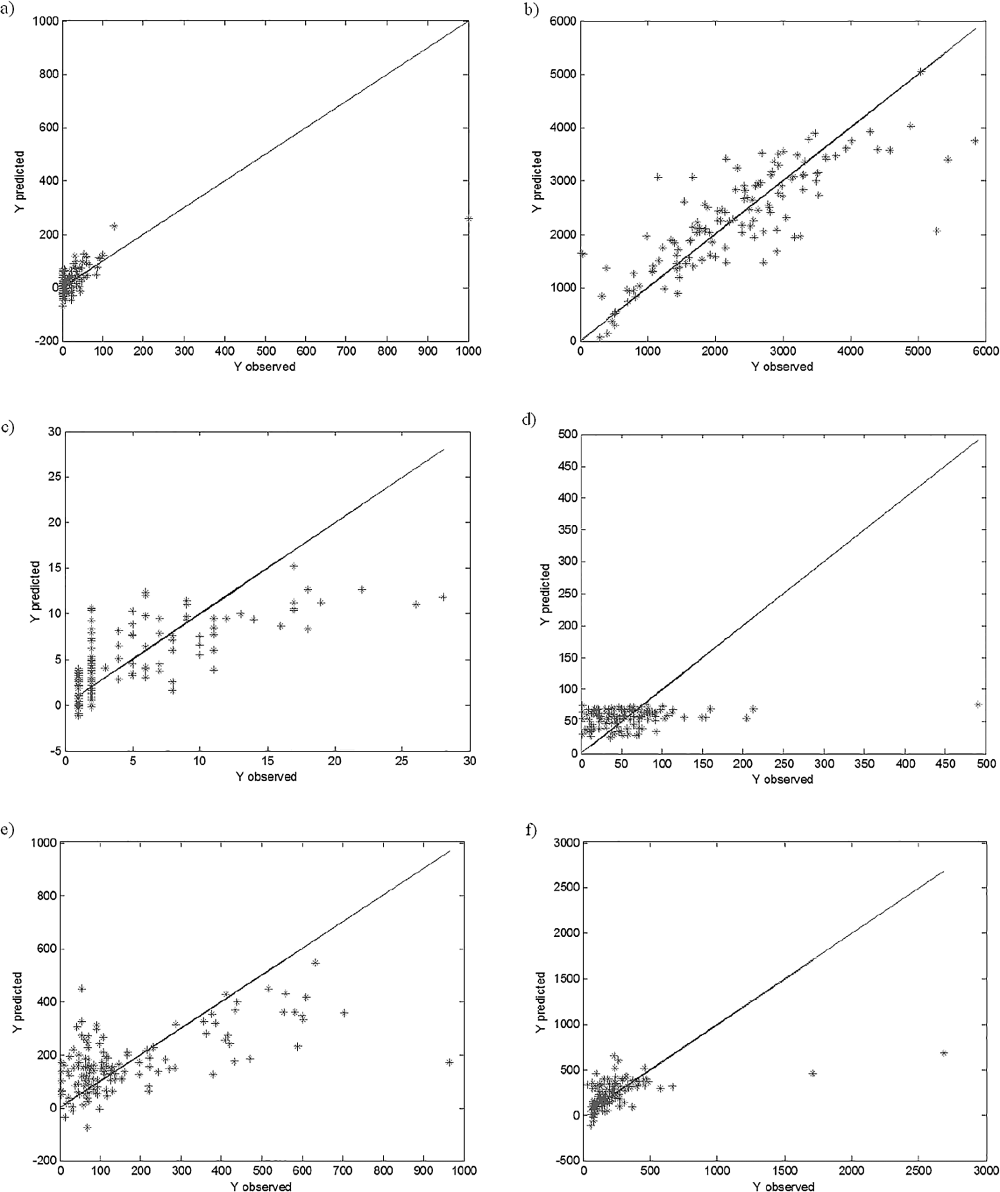
**PLS models of trace elements concentrations: (a) As, (b) Ba, (c) Cd, (d) Co, (e) Cr, (f) Cu, (g) Mn, (h) Ni, (i) Pb, (j) Rb, (k) Sr, (l) V and (m) Zn in** μ**g/g, based on the parameters specified in Table 1.**

The PLS method was applied to investigate the quantitative relationships between the concentrations of trace elements in coal and the remaining 24 parameters (see Table 1). The constructed PLS models are presented in Fig 2.

The correctly constructed PLS models need to be characterized by good fit and predictive abilities. These are assessed based on the values of the RMS and the RMSCV, respectively (see Table 2). In most of the constructed PLS models weak relationships between studied parameters were observed. Most of the constructed models were characterized by a weak fitting to the studied data and weak prediction abilities. The %RMS and %RMSCV errors were higher than 10% for eight out of thirteen PLS models constructed. Only the PLS models of concertation of As, Mn, Pb, Sr and Zn were characterized by relatively good fit and prediction abilities. This may be attributed to the presence of outliers in the studied data set, which can be clearly seen from Fig 2. In most cases some objects are located far away from the remaining ones, which results in the construction of incorrect models. As previously mentioned, the PLS as one of the Least Squares methods, is sensitive to the presence of outlying objects in the studied data. If the data set analyzed is contaminated with outliers, then even the PLS models for As, Mn, Pb, Sr and Zn concentrations are strongly influenced by the outliers.

**Table 2:** RMS and RMSCV errors of the constructed PLS models.

Trace element	Range, μg/g	Number of factors	RMS	RMSCV	%RMS	%RMSCV
As	[1–1000]	7	73.2317	90.1542	7.33	9.02
Ba	[14–5843]	2	664.8033	730.4704	11.41	12.53
Cd	[1–28]	10	4.0268	4.9249	14.91	18.24
Co	[1.5–490]	2	52.0733	53.6543	10.66	10.98
Cr	[3–965]	3	141.2543	157.5734	14.68	16.38
Cu	[35–2687]	3	238.9083	271.0099	9.01	10.22
Mn	[307–6568]	4	125.2308	191.4503	2.00	3.06
Ni	[5–1187]	2	130.2053	137.3366	11.02	11.62
Pb	[5–11099]	2	931.1152	980.1880	8.39	8.84
Rb	[3.5–253]	3	34.0336	38.7193	13.64	15.52
Sr	[148–4168]	9	166.5538	197.0697	4.41	4.90
V	[17–578]	4	54.4861	63.5758	9.71	11.33
Zn	[161–6793]	3	255.8820	301.3928	3.86	4.54

**Fig 3:**
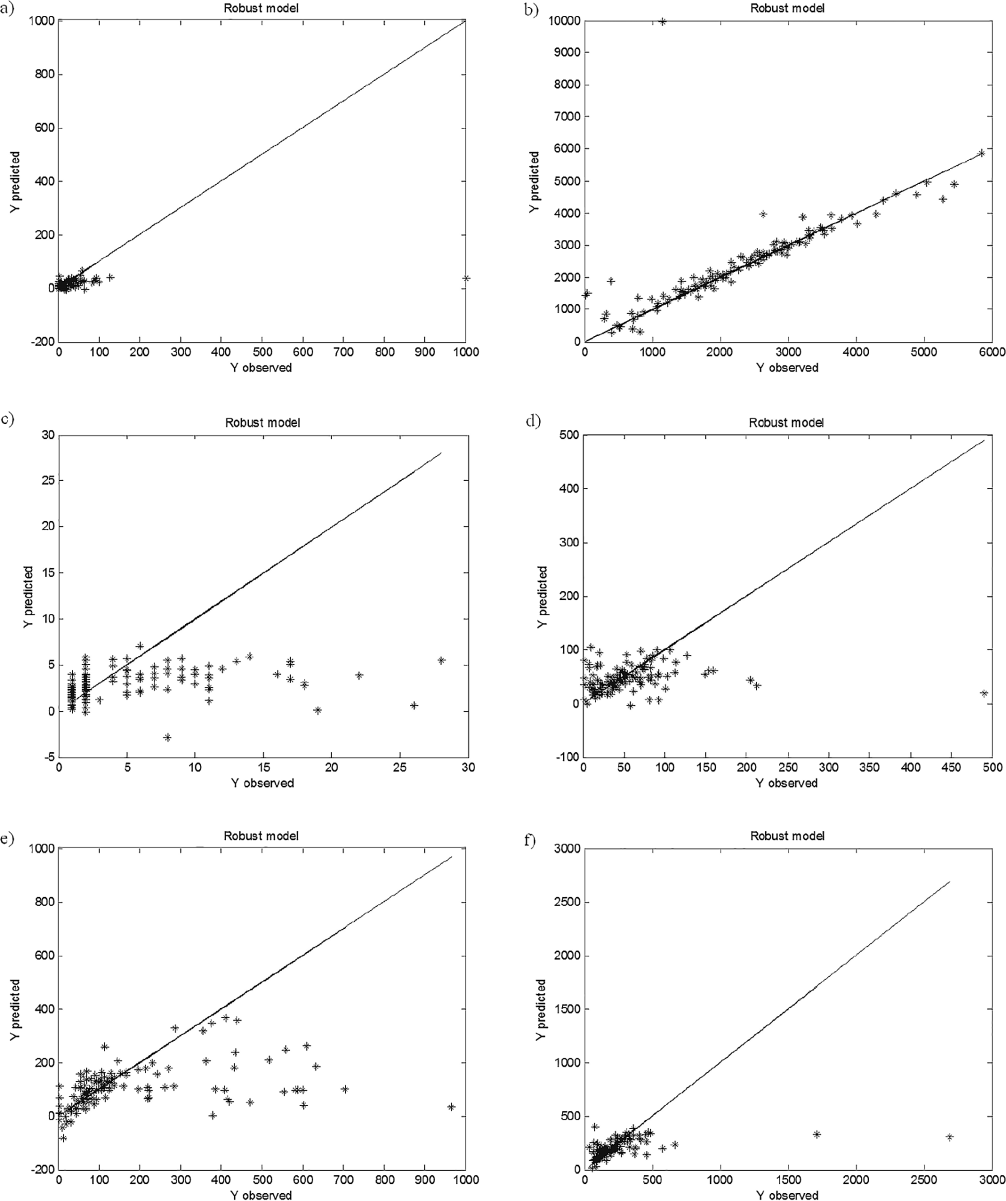
**rPLS models of trace elements concentrations: (a) As, (b) Ba, (c) Cd, (d) Co, (e) Cr, (f) Cu, (g) Mn, (h) Ni, (i) Pb, (j) Rb, (k) Sr, (l) V and (m) Zn in** μ**g/g, based on the parameters specified in Table 1, with the assumed fraction of outliers of 30%.**

The robust Partial Least Squares models were constructed since the initial PLS models were characterized by weak fit and predictive abilities. The rPLS models are constructed for the good majority of the data provided that the correct assumption of the data contamination is made. In the study presented 30% of data contamination was assumed for all the constructed robust PLS models presented in Fig 3.

**Fig 4:**
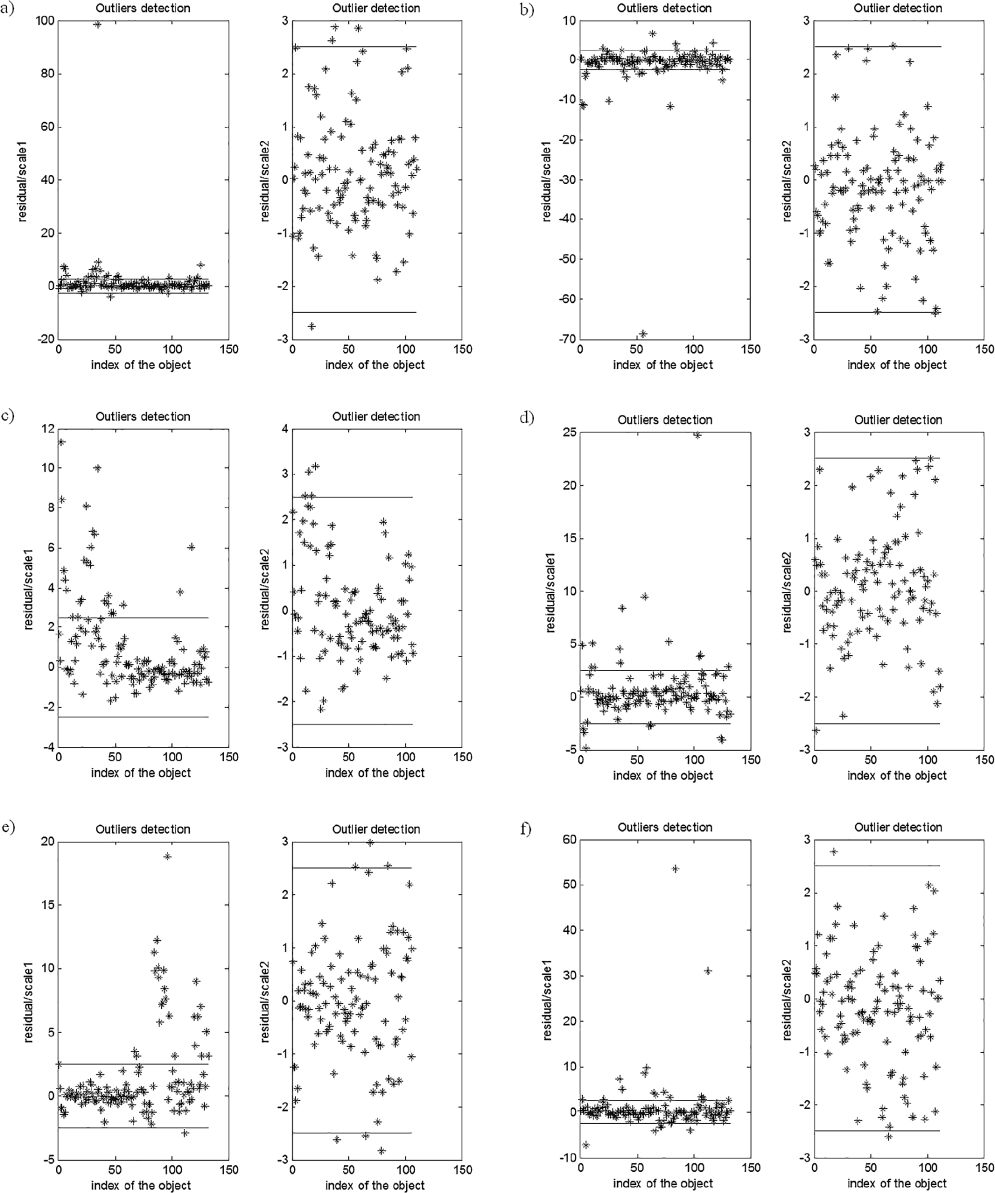
**Residuals plots: the first robust scale and the second robust scale for the robust PLS models of trace elements concentrations: (a) As, (b) Ba, (c) Cd, (d) Co, (e) Cr, (f) Cu, (g) Mn, (h) Ni, (i) Pb, (j) Rb, (k) Sr, (l) V and (m) Zn in** μ**g/g, based on the parameters specified in Table 1, with the assumed fraction of outliers of 30%.**

**Fig 5:**
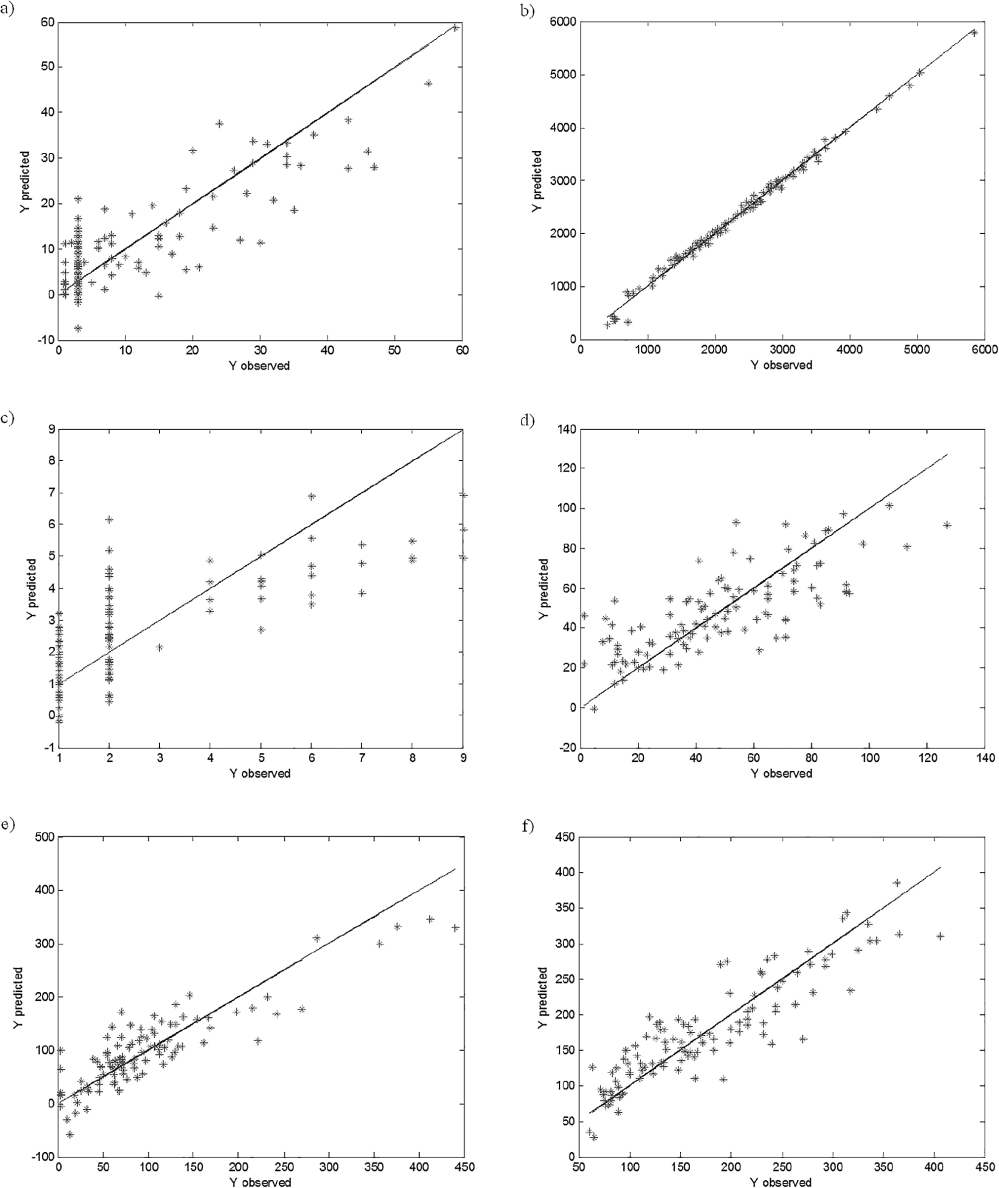
**PLS model of trace elements concentrations: (a) As, (b) Ba, (c) Cd, (d) Co, (e) Cr, (f) Cu, (g) Mn, (h) Ni, (i) Pb, (j) Rb, (k) Sr, (l) V and (m) Zn in** μ**g/g, based on the parameters specified in Table 1, after correct identification and elimination of outlying elements.**

The constructed robust PLS models were applied in a correct identification of outlying objects. The outliers were identified based on the robust scales, which means that all objects characterized by high residuals from the constructed robust PLS models were determined. The identified outliers were removed from the initial data set and the correct PLS models were constructed. The identification of the outliers in the constructed rPLS models based on the robust scales, as well as the final PLS models constructed after elimination of the outliers from the data set are presented in Figs 4 and 5, respectively.

The final PLS models constructed after elimination of the outlying objects were characterized by significantly improved fit and predictive abilities (see Fig 2). In Table 3 the values of RMS and RMSCV, as well as the objects identified as the outliers are presented.

**Table 3:** The RMS and RMSCV errors of the final PLS models.

Trace element	Range, μg/g	Objects identified as outliers	Number of factors	RMS	RMSCV	%RMS	%RMSCV
As	[1–59]	5; 6; 8; 27; 30; 31; 33; 34; 35; 36; 37; 42; 44; 46; 52; 53; 75; 97; 98; 116; 122; 123; 126	7	4.0136	5.1446	6.92	8.87
Ba	[413–5843]	3; 4; 5; 6; 21; 26; 39; 41; 53; 55; 56; 64; 69; 74; 80; 85; 112; 118; 125; 126	8	82.6789	102.5920	1.52	1.89
Cd	[1–9]	3; 4; 5; 6; 8; 13; 18; 19; 21; 23; 25; 26; 28; 29; 31; 32; 35; 38; 41; 44; 45; 47; 49; 58; 108; 118	9	1.0568	1.2376	13.21	15.47
Co	[1.5–124]	2; 3; 4; 5; 6; 10; 11; 13; 35; 36; 37; 57; 61; 62; 63; 78; 104; 105; 106; 124; 125; 131	4	10.9515	11.7845	8.94	9.62
Cr	[3–440]	1; 68; 69; 85; 86; 87; 88; 89; 90; 91; 92; 93; 94; 95; 96; 98; 101; 103; 112; 120; 121; 122; 123; 126; 127; 131; 132	4	28.8756	46.4559	6.61	10.63
Cu	[61–406]	2; 5; 21; 35; 37; 57; 59; 65; 66; 67; 71; 72; 74; 79; 80; 84; 97; 107; 108; 109; 113; 131	7	30.6015	31.8780	8.87	9.24
Mn	[307–6568]	25; 26; 28; 59; 73; 80; 85; 86; 87; 88	3	117.7068	181.5690	1.88	2.90
Ni	[5–409]	2; 5; 60; 76; 85; 86; 87; 88; 89; 91; 92; 96; 98; 107; 126; 127	7	39.5516	42.1372	9.79	10.43
Pb	[5–245]	1; 2; 5; 10; 11; 25; 27; 29; 33; 35; 36; 37; 99; 100; 105; 110; 111; 126	6	18.8880	19.344	7.87	8.06
Rb	[3.5–253]	5; 6; 24; 27; 33; 35; 38; 41; 42; 43; 44; 49; 52; 53; 54; 55; 56; 65; 70; 80; 112; 113; 114; 126; 130	4	19.8731	22.7238	7.97	9.11
Sr	[148–4168]	5; 26; 29; 43; 58; 65; 67; 74; 80; 85; 86; 87; 88; 89; 90; 97; 98; 111; 124; 128; 130	4	170.2257	186.5280	4.23	4.64
V	[17–478]	2; 5; 21; 37; 57; 58; 65; 66; 72; 75; 77; 79; 80; 93; 112; 121; 124; 125; 128; 129	6	27.7557	34.4384	6.02	7.47
Zn	[161–6793]	57; 62; 63; 73; 76; 85; 86; 87; 93; 94; 95; 96; 104; 105; 110; 127; 128; 129; 130; 131; 132	3	163.0831	210.3034	2.46	3.17

The RMS error for the final PLS models, except for the model of Cd concentration, was below 10%. It varied from 1.52% to 9.79% for the PLS models constructed for Ba and Ni, respectively. Only for the PLS constructed for Cd, the %RMS was 13.21%. Similarly, the prediction error (RMSCV) varied from 1.89% to 10.63% for the PLS models constructed for Ba and Cr, respectively. The uniqueness was observed in case of the Cd PLS model for which the value of RMSCV was 15.47%.

## Conclusions

1. The quantitative models of trace elements content (As, Ba, Cd, Co, Cr, Cu, Mn, Ni, Pb, Rb, Sr, V and Zn), based on the basic physical and chemical parameters of coal and coal ash components were constructed.
2. The initial PLS models were characterized by weak fitting to the studied data and weak prediction abilities. The reason for that was the presence of the outlying objects in the studied data set.
3. The construction of the robust PLS models and the correct identification of all outliers enabled the development of the final PLS models, characterized by significantly better fit and prediction abilities in comparison with the initial PLS models.
4. The fit error (%RMS) was below 10% for all the final PLS models constructed except for PLS model for Cd concentration. Only for three PLS models constructed (for concentrations of Cd, Cr and Ni) the prediction error (%RMSCV) was above 10%.

## Supporting Information

S1 Table Values of physical and chemical parameters of coal samples and coal ash components. (PDF)

S2 Table Concentrations of trace elements in coal samples. (PDF)
