# Supplementary material for: Quantitative Modelling of Trace Elements in Hard Coal
Source: PLoS One. 2016 Jul 20;11(7):e0159265. doi: 10.1371/journal.pone.0159265 (PMC4954660; doi:10.1371/journal.pone.0159265)
Supplement: S1 Table — (PDF) [file pone.0159265.s001.pdf]

Sample no														parameters										
	W	A	V	S	Qs	Qi	Ct	Sp	SiO2	Al2O3	Fe2O3	CaO	MgO	Na2O	K2O	SO3	TiO2	P2O5	BaO	Mn3O4	SrO	ZnO	Cl	F
1	4.43	26.09	25.03	0.89	28150	19646	55.34	0.66	56.19	17.76	8.64	5.74	3.1	0.55	2.27	4.19	0.76	0.09	0.08	0.1	0.045	0.11	0.044	0.012
2	5.58	4.62	32.02	0.57	29211	26471	73.4	0.35	31.6	17.64	14.63	14.65	5.46	1.14	0.94	11.77	0.77	0.26	0.14	0.18	0.12	0.25	0.042	0.005
3	7.28	2.5	33.29	0.56	28785	25660	72.4	0.25	22.58	14.93	17.7	17.3	5.13	2.04	0.37	17.74	0.96	0.199	0.18	0.179	0.139	0.154	0.032	0.005
4	7.8	2.68	33.24	0.55	28740	25859	71.9	0.21	23.2	15.56	17.61	16.78	4.84	2.11	0.37	17.52	0.99	0.185	0.187	0.184	0.142	0.152	0.032	0.004
5	6.11	10.18	35.56	0.69	27057	24530	67.09	0.27	41.75	14.43	13.27	10.63	5.38	1.26	1.68	9.35	0.75	0.315	0.037	0.912	0.033	0.078	0.06	0.008
6	5.18	27.39	34.71	1.06	21351	18873	53.08	0.63	50.46	15.58	11.4	7.09	4.67	0.91	2.11	6.01	0.79	0.244	0.033	0.192	0.018	0.024	0.15	0.015
7	4.38	18.28	33.92	0.8	24855	22464	62.13	0.33	48.44	22.67	9.5	5.27	3.83	0.65	2.7	5.1	1.02	0.108	0.139	0.112	0.053	0.068	0.152	0.017
8	3.67	23.84	33.34	0.92	22971	20340	57.71	0.44	52.39	23.64	8.79	3.66	3.19	0.58	2.93	2.82	1.06	0.135	0.161	0.09	0.045	0.079	0.122	0.017
9	4.83	7.62	32.84	0.76	28641	26347	70.91	0.25	33.88	22.08	11.71	11.36	4.99	1.14	1.5	11.7	0.9	0.267	0.183	0.134	0.176	0.101	0.134	0.01
10	4.01	6.87	33.5	0.8	29266	26992	72.65	0.32	30.76	20.18	12.46	12.99	5.5	1.33	1.25	13.23	0.88	0.24	0.206	0.146	0.184	0.127	0.134	0.01
11	4.72	4.51	33.93	0.7	29925	27903	74.03	0.22	30.85	19.84	14.68	11.42	5.31	1.38	1.18	13.33	0.84	0.124	0.268	0.165	0.189	0.089	0.12	0.009
12	4.81	7.16	32.76	0.66	28918	26713	72.09	0.28	31.96	19.28	11.03	13.86	6.05	1.46	1.43	12.8	1.05	0.129	0.163	0.152	0.137	0.068	0.175	0.009
13	3.84	3.39	34.46	0.59	30611	28629	76.21	0.19	17.05	14.45	17.84	19.46	7.72	2.34	0.6	18.95	0.5	0.072	0.282	0.221	0.179	0.14	0.213	0.01
14	2.43	3.99	30.47	0.22	31425	29565	78.97	0.04	19.07	15.96	21.02	17.33	9.3	2.29	0.82	12.48	0.4	0.189	0.227	0.19	0.153	0.029	0.299	0.009
15	2.44	4.66	31.04	0.22	31305	29188	78.29	0.03	28.62	15.57	17.38	15.91	8.39	2.17	0.91	9.11	0.74	0.181	0.192	0.134	0.119	0.032	0.326	0.008
16	2.11	4.13	31.64	0.24	31379	29558	78.48	0.02	26.59	17.6	15.22	17.73	8.4	2.12	1.04	9.46	0.71	0.177	0.253	0.122	0.156	0.044	0.306	0.007
17	2	4.7	31.75	0.24	31445	29434	78.4	0.02	28.89	16.67	16.32	16.22	8.55	1.87	1.12	8.07	0.91	0.167	0.194	0.117	0.12	0.328	0.326	0.007
18	2.23	18.19	31.58	0.28	26141	23489	65.32	0.03	51.77	20.81	9.35	5.29	3.93	0.82	2.41	2.53	1.01	0.129	0.079	0.078	0.045	0.02	0.372	0.014
19	2.64	6.75	31.28	0.46	30295	28472	75.44	0.1	27.3	17.16	13.65	17.53	7.89	1.16	1.36	11.37	0.67	0.675	0.227	0.15	0.237	0.099	0.159	0.012
20	2.31	7.36	31.28	0.51	30355	28371	75.24	0.09	34.88	18.51	10.99	13.48	6.2	1.25	1.45	9.62	0.89	1.292	0.271	0.097	0.352	0.161	0.189	0.014
21	2.18	3.92	31.5	0.47	31844	29722	78.91	0.1	20.49	16.78	15.52	17.63	7.6	2.38	1.26	14.39	0.51	2.06	0.449	0.146	0.465	0.08	0.185	0.013
22	2.49	7.07	31.04	0.5	30277	28122	75.39	0.12	24.88	13.93	13.72	21.18	9.33	1.16	1.13	11.97	0.84	0.742	0.24	0.169	0.217	0.046	0.157	0.011
23	2.79	9.09	30.22	0.52	29583	27291	72.54	0.05	34.92	19.2	11.2	13.18	6.34	1.12	1.7	9.16	0.84	0.798	0.233	0.118	0.261	0.187	0.191	0.011
24	2.23	15.15	31.53	0.69	27372	24451	67.86	0.31	44.74	19.37	10.41	8.65	4.92	0.78	2.21	6.46	0.87	0.366	0.363	0.106	0.097	0.139	0.204	0.013
25	5.98	6.3	33.27	0.7	28336	26480	76.7	0.34	26.33	19.73	13.12	16.28	6.66	0.77	0.92	12.31	0.79	1.83	0.333	0.086	0.369	0.161	0.0025	0.013
26	4.21	3.27	32.03	0.5	30911	33410	77.28	0.1	20.04	15.14	14.32	19.32	8.03	1.39	0.75	18.99	0.45	0.127	0.53	0.109	0.242	0.058	0.01	0.009
27	5.76	8.59	32.25	0.85	27612	25379	69.28	0.47	28.19	20.76	11.76	15.31	6.23	0.73	1.05	12.08	0.89	1.71	0.271	0.07	0.364	0.116	0.025	0.013
28	2.73	3.75	32.86	0.61	31122	29006	77.35	0.22	16.04	12.71	16.83	22.82	9.64	0.98	0.52	19.11	0.49	0.13	0.295	0.128	0.201	0.04	0.015	0.007
29	6	8.94	31.32	0.83	27134	24839	68.29	0.45	28.88	21.15	12.52	14.86	6.26	0.62	1.33	10.98	0.9	1.45	0.195	0.093	0.19	0.098	0.025	0.014
30	4.24	4.23	31.98	0.58	30409	28336	75.85	0.16	22.82	17.18	16.29	16.02	7.2	1.07	0.97	16.52	0.69	0.113	0.286	0.12	0.152	0.057	0.021	0.006
31	6.49	10.23	32.95	0.87	26429	24073	66.85	0.45	32.77	22.15	12.04	12.49	5.42	0.61	1.53	9.97	0.97	1.05	0.156	0.075	0.223	0.112	0.025	0.015
32	3.43	6.16	32.22	0.58	30437	28160	75.75	0.19	33.61	22.49	12.23	10.98	5.34	0.83	1.47	10.59	0.97	0.102	0.232	0.09	0.145	0.037	0.023	0.008
33	7.03	17.17	31.85	1	23398	21024	59.15	0.53	43.53	22.99	8.95	8.55	4.32	0.58	2.03	6.31	0.95	0.95	0.099	0.058	0.101	0.088	0.0025	0.018
34	2.75	20.32	31.86	0.74	24770	22467	61.97	0.34	53.06	20.24	9.86	4.86	3.5	0.56	2.44	3.94	0.83	0.073	0.089	0.065	0.032	0.027	0.047	0.014
35	1.33	20.84	27.58	1.16	27072	25065	64.6	0.58	45.88	24.09	8.44	4.49	3.97	1	3.17	4.42	1.06	0.56	0.341	0.081	0.078	0.314	0.265	0.021
36	1.5	26.36	25.89	1.21	24644	21843	59.57	0.62	47.22	24.15	8.1	4.59	3.78	0.89	3.23	5.12	1.05	0.46	0.354	0.077	0.075	0.088	0.272	0.022
37	1.53	5.76	31.09	0.7	32691	30380	78.28	0.28	35.92	25.23	11.49	7.51	4.17	1.36	1.7	7.38	1.26	2.16	0.546	0.079	0.295	0.064	0.179	0.019
38	2.19	3.57	33.38	0.79	32287	30041	79.98	0.79	17.63	12.65	25.76	16.08	8.31	1.01	0.77	15.13	0.55	0.616	0.272	0.247	0.15	0.143	0.042	0.0075
39	2.5	4.09	32.51	0.81	32087	30423	79.97	0.4	13.48	9.64	26.34	18.15	7.67	0.95	0.5	21.3	0.48	0.604	0.206	0.289	0.114	0.051	0.046	0.0057
40	2.69	4.75	32.61	0.66	31726	29674	78.94	0.3	28.26	15.88	17.51	13.39	6.37	0.88	1.13	14.21	0.85	0.532	0.21	0.169	0.145	0.105	0.048	0.0065
41	2.36	18.96	32	1.28	26363	23900	66.1	0.75	48.12	17.54	12.11	6.64	4.06	0.54	1.98	7.47	0.83	0.272	0.088	0.125	0.034	0.037	0.247	0.0125
42	1.52	8.5	30.62	0.67	31578	29152	77.89	0.18	34.93	22.88	13.54	8.45	4.89	1.49	1.73	7.62	0.85	1.71	0.301	0.107	0.262	0.777	0.055	0.0129
43	2.12	29.97	29.65	0.83	22452	19635	56.61	0.27	51.57	27.02	7.21	2.96	2.85	0.68	2.92	2.37	1.02	0.447	0.164	0.043	0.064	0.142	0.13	0.0217
44	1.27	7.29	29.37	0.62	32286	29990	80.14	0.09	43.94	26.07	9.7	5.53	3.69	1.03	2.17	5.2	0.95	0.448	0.172	0.062	0.12	0.156	0.17	0.0117
45	1.18	4.09	31.17	0.61	33433	31932	82.97	0.1	19.16	13.5	16.51	20.04	7.68	1.64	0.46	17.32	0.8	1.72	0.328	0.18	0.286	0.026	0.1354	0.0091
46	1.29	4.89	31.14	0.57	32955	31426	81.72	0.09	30.12	26.32	9.81	11.52	4.25	1.49	1.07	8.42	1.38	4.16	0.563	0.059	0.493	0.021	0.121	0.0128
47	1.47	6	30.93	0.61	32526	30624	80.42	0.08	35.93	24.21	10.01	9.54	4.19	1.21	1.38	8.33	1.37	2.49	0.378	0.067	0.27	0.02	0.128	0.0125
48	1.53	5.78	31	0.62	32532	30569	80.47	0.05	37.92	26.41	9.44	8.5	3.64	1.11	1.6	6.08	1.38	2.8	0.388	0.05	0.269	0.03	0.127	0.0128
49	1.61	24.99	30.69	0.8	24832	22761	62.34	0.31	49.56	23.81	8.33	5.26	3.24	0.74	2.49	3.77	1.17	0.91	0.131	0.053	0.072	0.024	0.177	0.0185
50	1.18	4.42	30.04	0.51	33344	31868	83.04	0.09	19.63	16.12	21.3	15.12	7.36	1.68	0.7	15.35	0.65	1.15	0.423	0.219	0.209	0.044	0.201	0.0087
51	1.13	4.34	29.87	0.54	33478	31963	83.2	0.12	19.55	16.01	18.52	16.53	7.88	1.76	0.7	15.75	0.67	1.36	0.439	0.199	0.25	0.035	0.195	0.0075
52	1.44	7.4	30.76	0.54	32041	30446	79.21	0.18	32.33	23.43	11.41	10.45	5.24	1.75	1.47	10.03	0.96	1.53	0.371	0.086	0.276	0.066	0.255	0.0108
53	1.52	17.7	31.35	0.65	27913	25311	68.91	0.27	43.17	23.17	9.39	6.56	4.51	1.48	2.55	6.2	0.93	0.696	0.159	0.06	0.077	0.054	0.564	0.0157
54	1.34	6.36	31.12	0.67	32541	30897	80.79	0.26	28.96	20.23	12.32	13.06	6	1.6	1.24	13.03	0.84	1.65	0.329	0.095	0.25	0.027	0.224	0.0142
55	1.16	20.63	31.78	0.62	26729	24229	66.41	0.22	44.83	24.13	8.68	6.04	4.33	1.44	2.62	5.39	0.96	0.72	0.18	0.053	0.087	0.043	0.529	0.0172
56	1.29	26.01	31.2	0.62	24589	22672	61.05	0.23	48.22	24.04	8.27	4.76	3.9	0.95	2.86	4.76	0.93	0.046	1.29	0.046	0.053	0.044	0.389	0.0199
57	6.46	4.04	31.18	0.33	28933	26274	72.3	0.15	11.98	9.41	12.46	33.63	11.07	1.45	0.24	18.61	0.31	0.13	0.19	0.16	0.18	0.042	0.153	0.003

**58**	6.43	6.02	30.68	0.7	28267	25595	70.94	0.41	18.18	9.63	19.83	22.07	9.27	1.18	0.36	18.01	0.58	0.13	0.33	0.17	0.08	0.031	0.1	0.001
**59**	7.16	3.29	31.31	0.33	29036	24135	72.96	0.13	15.07	12.99	12.17	27.81	10.79	2.33	0.42	16.75	0.43	0.14	0.24	0.17	0.19	0.03	0.137	0.002
**60**	5.86	2.7	32.83	0.42	29902	25722	74.51	0.19	16.43	13.77	10.84	20.52	10.4	2.35	0.31	23.69	0.25	0.23	0.24	0.18	0.23	0.19	0.173	0.002
**61**	6.37	2.87	32.75	0.44	29737	25415	74.81	0.31	14.78	12.91	10.68	22.05	11.26	2.08	0.23	24.48	0.22	0.18	0.21	0.18	0.23	0.11	0.148	0.001
**62**	6.39	2.8	32.72	0.4	29729	25717	74.57	0.17	14.45	12.38	11.05	21.73	11.26	1.88	0.26	25.46	0.22	0.17	0.2	0.18	0.21	0.029	0.12	0.001
**63**	6.33	2.73	32.77	0.41	29812	25792	74.22	0.2	15.52	13.33	10.8	20.27	10.6	2	0.23	25.85	0.23	0.17	0.22	0.16	0.21	0.031	0.137	0.001
**64**	4.53	25.45	26.17	0.94	22329	20558	55.86	0.75	51.63	26.95	7.14	3.1	2.8	0.46	2.98	2.04	1.24	0.36	0.59	0.1	0.1	0.024	0.027	0.016
**65**	3.6	31.11	24.38	0.89	20688	18604	50.94	0.89	53.29	25.29	6.88	3.05	2.66	0.46	3.04	2.37	1.53	0.48	0.12	0.11	0.153	0.15	0.047	0.016
**66**	4.24	7.5	26.91	0.65	29698	26849	73.04	0.27	40.1	27.66	9.35	7	4.04	0.79	1.58	5.32	0.93	1.75	0.41	0.12	0.38	0.12	0.151	0.008
**67**	3.6	31.11	24.38	0.89	20688	18604	50.94	0.58	53.29	25.29	6.88	3.05	2.66	0.46	3.04	2.37	1.53	0.48	0.12	0.11	0.146	0.15	0.047	0.016
**68**	3.42	6.52	32.92	0.81	30227	26714	74.33	0.34	27.8	23.89	12.31	12.79	5.96	0.57	1.13	11.03	0.72	2.46	0.31	0.19	0.28	0.02	0.084	0.01
**69**	3.6	7.92	32.36	0.84	29583	26968	73.42	0.45	29.49	23.4	11.95	11.75	5.85	0.56	1.32	11.65	0.72	2.27	0.26	0.19	0.23	0.019	0.102	0.012
**70**	3.38	17.59	33.26	0.6	26266	22842	62.67	0.29	54.97	22.8	6.45	3.81	3.06	0.45	2.87	2.8	1.05	0.72	0.17	0.11	0.15	0.043	0.1	0.008
**71**	3.47	6	33.27	0.58	30527	27783	73.58	0.25	37.56	26.17	9.82	8.38	4.57	0.58	1.47	7.29	0.98	1.59	0.39	0.17	0.3	0.046	0.038	0.004
**72**	3.68	7.01	33.61	0.58	29879	28053	72.21	0.2	30.22	25.02	8.91	12.71	6.65	0.44	0.82	10.52	0.74	2.26	0.49	0.24	0.44	0.044	0.044	0.008
**73**	4.82	6.08	33.98	0.68	29913	27028	72.23	0.36	30.28	25.97	10.49	11.08	5.66	0.59	1.03	9.78	0.83	2.59	0.51	0.15	0.39	0.11	0.076	0.004
**74**	3.6	3.51	30.61	0.42	31608	29248	79.62	0.22	18.01	13.94	11.44	18.11	10.45	3.14	0.61	22.75	0.43	0.07	0.11	0.25	0.184	0.34	0.317	0.004
**75**	3.33	3.41	31.06	0.41	31788	29192	79.48	0.19	21.87	14.07	12.88	15.31	8.8	4.03	0.84	20.34	0.5	0.15	0.15	0.27	0.13	0.19	0.346	0.001
**76**	3.25	3.26	33.2	0.39	31813	29384	78.99	0.15	19.29	10.23	10.4	21.88	12.71	2.64	0.84	20.56	0.33	0.12	0.29	0.22	0.16	0.052	0.301	0.002
**77**	3.73	3.68	31.92	0.53	31802	29494	78.57	0.25	24.79	14.74	14.97	14.91	8.34	2.9	0.97	15.63	0.87	0.14	0.28	0.27	0.17	0.63	0.31	0.002
**78**	3.21	3.34	33.34	0.54	32305	29600	79.73	0.2	22.48	13.92	18.92	14.71	7.54	2.69	0.83	16.46	0.74	0.24	0.31	0.43	0.23	0.1	0.304	0.003
**79**	3.8	2.14	30.84	0.33	31957	29652	80.39	0.02	23.52	16.99	12.12	14	8.2	4.53	1.09	18.07	0.44	0.07	0.15	0.26	0.13	0.042	0.255	0.004
**80**	3.54	20.53	26.67	0.6	25151	22431	61.82	0.39	55.34	17.23	9.11	5.01	4.21	1.09	2.3	4.02	0.91	0.08	0.22	0.16	0.164	0.043	0.277	0.007
**81**	3.27	3.08	31.51	0.5	32167	29568	78.6	0.22	12.01	10.6	18.38	18.58	9.74	2.82	0.58	25.54	0.31	0.74	0.33	0.23	0.23	0.55	0.264	0.003
**82**	3.19	2.65	31.72	0.45	32246	29513	78.43	0.23	16.24	13.6	17.63	15.94	8.49	3.21	0.68	21.15	0.41	0.9	0.35	0.2	0.33	0.42	0.223	0.002
**83**	2.82	2.87	31.52	0.42	32130	29434	78.13	0.23	15.46	11.95	17.09	17.25	9.55	2.93	0.77	22.84	0.42	0.48	0.31	0.2	0.19	0.23	0.246	0.002
**84**	3.43	3.22	30.22	0.35	31652	29315	78.56	0.15	20.01	15.77	11.31	19.08	10.65	2.65	0.82	17.37	0.49	0.09	0.25	0.22	0.12	0.59	0.257	0.003
**85**	3.74	9.69	31.47	0.44	29248	26423	70.96	0.25	41.36	12.47	13.03	11.89	7.17	1.73	1.4	9.54	0.49	0.11	0.005	0.25	0.005	0.005	0.365	0.015
**86**	3.52	12.54	32.08	0.49	28318	25519	68.3	0.27	48.05	14.86	10.89	9.04	5.53	1.51	1.69	6.82	0.64	0.14	0.005	0.22	0.005	0.005	0.365	0.011
**87**	3.49	16.46	31.3	0.62	26908	24321	65.17	0.33	53.97	13.28	9.69	8.36	5.16	1.24	1.54	6.33	0.57	0.11	0.005	0.18	0.005	0.005	0.35	0.008
**88**	2.89	19.21	32.02	0.47	26182	23395	63.17	0.27	54.74	16.09	9.16	6.22	4.38	1.12	1.94	4.72	0.74	0.15	0.01	0.18	0.005	0.005	0.35	0.01
**89**	3.15	21.37	31.56	0.49	25343	22632	61.68	0.23	56.73	15.87	8.8	5.55	3.95	1.05	2.07	4.36	0.71	0.16	0.01	0.18	0.005	0.005	0.427	0.011
**90**	2.55	25.29	34.1	0.6	24253	21581	58.79	0.31	53.74	19.34	10.52	4.05	3.45	0.95	2.53	3.46	0.98	0.27	0.01	0.21	0.005	0.005	0.239	0.016
**91**	2.79	29.08	34.53	0.47	22885	20227	54.93	0.16	54.98	20.06	9.73	3.54	3.25	0.99	2.7	2.6	1.03	0.28	0.02	0.2	0.02	0.005	0.239	0.02
**92**	2.81	31.26	33.16	0.39	21948	19571	53.19	0.2	58.49	19.39	7.93	3.26	3.25	0.87	2.51	2.28	1	0.2	0.01	0.15	0.01	0.01	0.181	0.016
**93**	3.5	3.59	32.77	0.44	31993	29661	78.46	0.16	29.31	14.71	16.68	13.3	7.45	1.61	1.35	13.51	0.68	1.1	0.28	0.27	0.26	0.098	0.21	0.006
**94**	4.04	3.25	30	0.55	31849	29835	78.57	0.29	17.68	9.18	15.03	18.73	11	2.96	0.91	22.72	0.35	0.22	0.27	0.18	0.1	0.094	0.216	0.001
**95**	3.85	3	30.96	0.47	31845	29854	78.7	0.26	11.01	8.16	15.36	21.45	12.26	2.75	0.71	26.52	0.32	0.11	0.3	0.19	0.1	0.099	0.189	0.001
**96**	3.93	2.9	31.51	0.5	31915	29804	79.91	0.27	16.02	9.83	13.46	19.37	11.01	2.97	1	24.34	0.36	0.22	0.32	0.2	0.12	0.089	0.273	0.006
**97**	3.76	21.81	27.33	0.67	23964	20964	59.87	0.14	47.31	22.86	8.09	6.42	4.98	1.1	2.34	4.42	1.02	0.23	0.3	0.13	0.116	0.14	0.267	0.011
**98**	3.92	12.44	30.62	0.52	27981	24853	69.17	0.26	38.73	17.2	13.79	10.31	6.51	1.51	2.21	7.74	0.71	0.15	0.16	0.24	0.121	0.12	0.318	0.009
**99**	3.3	3.66	30.25	0.32	31867	29519	78.16	0.11	29.67	20.42	9.63	13.24	7.59	3.87	1.13	12.31	0.7	0.1	0.2	0.17	0.11	0.024	0.179	0.001
**100**	3.34	4.1	29.75	0.33	31691	29403	77.59	0.1	32.71	22.74	10.14	10.87	6.42	3.5	1.16	10.18	0.86	0.1	0.19	0.17	0.11	0.022	0.193	0.001
**101**	3.18	3.33	30.24	0.29	32139	30022	78.43	0.12	24.5	16.52	10.85	17.07	8.74	3.76	0.83	15.79	0.5	0.11	0.21	0.21	0.11	0.02	0.14	0.006
**102**	3.35	3.59	30.1	0.31	31900	29688	77.96	0.11	29.12	20.51	10.43	13.75	7.66	3.85	0.96	11.72	0.64	0.09	0.21	0.22	0.12	0.025	0.148	0.004
**103**	3.22	4.34	30	0.3	31577	29448	77.51	0.11	25.38	15.24	10.27	19.89	10.41	3.11	0.57	13.25	0.64	0.09	0.18	0.21	0.1	0.025	0.227	0.003
**104**	2.12	4.51	29.4	0.45	32497	30759	82.16	0.18	9.42	7.81	19.83	26.84	9.82	1.45	0.4	22.41	0.34	0.28	0.33	0.43	0.2	0.021	0.195	0.001
**105**	2.23	4.37	29.54	0.44	32489	30705	81.57	0.18	14.59	11.83	17.94	22.67	9.51	1.65	0.72	18.83	0.49	0.3	0.37	0.37	0.21	0.023	0.233	0.001
**106**	2.14	4.23	29.4	0.4	32707	31023	81.9	0.14	8.65	8.17	22.12	27.16	10.62	1.16	0.42	19.76	0.36	0.22	0.29	0.46	0.18	0.022	0.244	0.001
**107**	2.62	4.31	29.47	0.46	32568	30597	81.69	0.12	26.66	21.64	14.63	13.48	6.77	1.69	1.56	10.79	0.88	0.32	0.37	0.24	0.26	0.019	0.179	0.002
**108**	2.56	4.66	29.22	0.45	32428	30357	81.18	0.15	28.43	22.82	13.8	14.03	6.43	1.6	1.51	8.75	0.94	0.31	0.3	0.24	0.24	0.02	0.197	0.002
**109**	2.59	4.56	29.23	0.46	32399	30385	80.97	0.18	26.68	21.03	15.11	14.52	7.25	1.7	1.48	9.7	0.9	0.29	0.29	0.25	0.24	0.022	0.211	0.003
**110**	2.42	20.89	25.23	0.78	26097	23616	65.66	0.26	44.79	24.38	9.48	7.39	4.21	0.95	2.35	4.22	1.02	0.16	0.22	0.15	0.11	0.018	0.21	0.008
**111**	2.4	11.96	27.54	0.6	29568	27280	73.82	0.29	49.63	24.42	8.14	5.63	3.5	0.76	2.56	3.33	1.01	0.12	0.19	0.13	0.142	0.02	0.193	0.006
**112**	1.88	6.16	29.13	0.59	32050	30003	77.02	0.25	37.97	33.57	7.89	5.39	3.06	1.54	1.61	4.82	1.36	0.35	0.48	0.11	0.46	0.019	0.133	0.008
**113**	1.95	6.63	28.61	0.6	32129	30321	79.75	0.26	34.34	29.64	9.72	7.84	4.06	1.37	1.48	8.08	1.05	0.88	0.26	0.16	0.27	0.14	0.254	0.004
**114**	3.64	10.35	30.68	0.73	28794	25217	72.27	0.39	44.07	10.07	10.89	15.03	6.33	0.83	0.81	10.54	0.67	0.1	0.164	0.185	0.065	0.081	0.145	0.008
**115**	3.74	11.94	30.26	0.66	27970	25104	70.23	0.33	46.42	16.04	10.29	10.31	4.99	0.58	1.26	8.5	1.09	0.08	0.121	0.166	0.041	0.081	0.113	0.01
**116**	3.24	11.92	30.73	0.64	28546	25644	71.15	0.27	46.71	13.23	10.33	11.74	4.34	1.09	1.15	8.67	0.95	0.057	0.16	0.141	0.061	0.057	0.347	0.009

**117**	3.19	4.5	33.2	0.5	31349	28698	78.08	0.14	25.26	17.45	11.36	18.07	7.64	1.5	0.87	15.27	0.93	0.178	0.394	0.277	0.239	0.216	0.121	0.006
**118**	3.03	3.22	33.15	0.39	31928	29537	79.35	0.07	21.7	16.62	12.85	18.85	8.17	2.67	0.84	15.08	0.94	0.065	0.607	0.176	0.292	0.846	0.26	0.006
**119**	3.54	4.01	31.67	0.52	31340	28953	78.47	0.21	18.6	11.07	17.08	20.92	8.99	2.17	0.55	18.23	1.11	0.074	0.314	0.226	0.15	0.274	0.247	0.008
**120**	1.33	3.9	29.55	0.49	33821	31876	83.77	0.3	34.42	21	23.28	5.01	3.77	2.12	2.04	5.83	0.9	0.14	0.35	0.39	0.11	0.022	0.226	0.004
**121**	1.5	4.65	29.8	0.57	33322	31143	82.57	0.19	38.25	23.63	19.55	4.61	3.33	1.77	1.79	4.51	1.03	0.21	0.29	0.33	0.1	0.026	0.222	0.004
**122**	1.51	3.26	30.16	0.55	33943	31902	84.54	0.25	13.65	10.83	42.4	9.37	5.81	1.98	0.61	12.76	0.38	0.23	0.65	0.73	0.13	0.025	0.226	0.001
**123**	1.36	4.62	29.81	0.49	33446	31775	84.02	0.23	30.22	17.63	27.99	6.65	4.66	1.78	1.45	7.16	0.83	0.16	0.37	0.52	0.1	0.025	0.2	0.002
**124**	2.89	3.79	30.3	0.55	32010	30093	79.83	0.28	35.59	25.07	17.17	4.89	2.59	3.84	1.58	4.88	1.04	1.66	0.34	0.13	0.38	0.024	0.24	0.004
**125**	3.47	5.3	30.1	0.87	31359	29476	76.71	0.61	31.78	24.08	20.58	6.36	2.63	3.65	1.36	4.83	1.35	2.2	0.24	0.11	0.32	0.023	0.328	0.007
**126**	3.19	5.72	31.42	1.36	31300	29714	76.27	1.04	19.85	17.29	26.43	11.61	5.76	1.78	0.46	12.49	0.64	2.09	0.36	0.24	0.21	0.26	0.257	0.006
**127**	2.34	3.84	30.85	0.68	32417	30113	77.87	0.28	25.12	19.51	16.21	11.63	6.62	2.92	0.79	13.28	0.72	1.49	0.41	0.35	0.31	0.144	0.261	0.004
**128**	2.25	3.39	30.31	0.6	32334	30178	79.2	0.29	35.13	23.24	17.28	5.67	3.47	4.81	1.29	5.89	0.82	0.96	0.32	0.23	0.27	0.164	0.272	0.003
**129**	1.98	4	31.06	0.55	32363	30044	77.9	0.3	40.05	26	13.36	4.93	3.04	3.31	1.66	4.02	1.08	1.15	0.32	0.14	0.35	0.148	0.286	0.004
**130**	2.2	3.82	30.54	0.46	32320	30022	77.61	0.2	51.79	22.45	11.66	2.61	1.46	3.12	1.56	1.79	1.03	1.25	0.19	0.1	0.4	0.155	0.268	0.005
**131**	1.3	3.26	31.93	0.38	33818	30944	81.41	0.1	33.78	19.53	14.69	9.21	5.77	2.15	1.22	10.93	1.36	0.14	0.39	0.17	0.2	0.146	0.177	0.002
**132**	1.43	3.58	32.29	0.32	33737	31859	80.21	0.05	24.75	15.64	15.37	14.37	8.56	1.49	0.99	16.24	1.07	0.06	0.39	0.19	0.17	0.146	0.093	0.002
