# Supplementary material for: Quantitative Modelling of Trace Elements in Hard Coal
Source: PLoS One. 2016 Jul 20;11(7):e0159265. doi: 10.1371/journal.pone.0159265 (PMC4954660; doi:10.1371/journal.pone.0159265)
Supplement: S2 Table — (PDF) [file pone.0159265.s002.pdf]

| Sample no | Hg | As | Ba | Cd | Co | Cr | Cu | Mn | Ni | Pb | Rb | Sb | Sr | V | Zn |
|---|---|---|---|---|---|---|---|---|---|---|---|---|---|---|---|
| **1** | 0.04 | 3 | 723 | 6 | 49 | 222 | 132 | 715 | 77 | 301 | 203 | 1.5 | 266 | 220 | 900 |
| **2** | 0.04 | 3 | 1227 | 2 | 154 | 126 | 332 | 1292 | 392 | 512 | 78 | 8 | 1006 | 361 | 1994 |
| **3** | 0.03 | 3 | 14 | 26 | 8 | 54 | 200 | 1286 | 46 | 164 | 11 | 1.5 | 1177 | 117 | 1241 |
| **4** | 0.03 | 21 | 48 | 19 | 8 | 66 | 208 | 1325 | 47 | 171 | 19 | 5 | 1202 | 120 | 1225 |
| **5** | 0.03 | 66 | 327 | 8 | 10 | 54 | 77 | 6568 | 48 | 111 | 18 | 5 | 278 | 147 | 623 |
| **6** | 0.09 | 84 | 297 | 11 | 1.5 | 43 | 61 | 1380 | 27 | 24 | 47 | 4 | 148 | 157 | 193 |
| **7** | 0.03 | 28 | 1245 | 2 | 47 | 113 | 94 | 806 | 83 | 176 | 161 | 1.5 | 445 | 268 | 548 |
| **8** | 0.03 | 66 | 1442 | 11 | 51 | 118 | 90 | 645 | 75 | 175 | 194 | 1.5 | 381 | 280 | 637 |
| **9** | 0.03 | 3 | 1635 | 2 | 92 | 112 | 160 | 965 | 143 | 140 | 148 | 1.5 | 1485 | 318 | 813 |
| **10** | 0.03 | 3 | 1847 | 2 | 103 | 137 | 170 | 1050 | 186 | 258 | 122 | 1.5 | 1554 | 290 | 1022 |
| **11** | 0.03 | 15 | 2402 | 2 | 149 | 122 | 183 | 1186 | 233 | 325 | 106 | 15 | 1598 | 260 | 712 |
| **12** | 0.03 | 3 | 1462 | 5 | 60 | 111 | 165 | 1094 | 101 | 189 | 125 | 1.5 | 1158 | 257 | 546 |
| **13** | 0.11 | 3 | 2527 | 9 | 88 | 72 | 199 | 1590 | 132 | 140 | 49 | 1.5 | 1517 | 115 | 1127 |
| **14** | 0.03 | 3 | 2037 | 2 | 15 | 25 | 216 | 1368 | 108 | 24 | 22 | 13 | 1295 | 17 | 229 |
| **15** | 0.03 | 3 | 1720 | 4 | 15 | 47 | 148 | 965 | 114 | 14 | 12 | 27 | 1009 | 82 | 258 |
| **16** | 0.03 | 3 | 2268 | 7 | 13 | 57 | 263 | 880 | 142 | 7 | 30 | 18 | 1315 | 83 | 356 |
| **17** | 0.03 | 8 | 1740 | 6 | 24 | 62 | 244 | 842 | 122 | 14 | 30 | 25 | 1018 | 139 | 2635 |
| **18** | 0.03 | 27 | 704 | 8 | 16 | 70 | 123 | 560 | 53 | 35 | 88 | 15 | 378 | 211 | 161 |
| **19** | 0.17 | 15 | 2035 | 12 | 19 | 45 | 232 | 1080 | 84 | 105 | 65 | 26 | 2002 | 137 | 798 |
| **20** | 0.06 | 15 | 2423 | 9 | 24 | 61 | 271 | 699 | 91 | 88 | 103 | 23 | 2979 | 213 | 1293 |
| **21** | 0.03 | 3 | 4021 | 13 | 18 | 39 | 276 | 1051 | 115 | 112 | 61 | 20 | 3931 | 55 | 643 |
| **22** | 0.28 | 3 | 2146 | 2 | 14 | 21 | 244 | 1220 | 82 | 114 | 40 | 29 | 1838 | 131 | 371 |
| **23** | 0.08 | 30 | 2089 | 16 | 25 | 50 | 216 | 851 | 85 | 80 | 102 | 26 | 2204 | 208 | 1499 |
| **24** | 0.15 | 23 | 3251 | 8 | 13 | 63 | 192 | 761 | 63 | 74 | 81 | 20 | 820 | 188 | 1117 |
| **25** | 0.03 | 43 | 2981 | 22 | 45 | 59 | 100 | 616 | 107 | 684 | 47 | 13 | 3118 | 147 | 1297 |
| **26** | 0.06 | 3 | 2640 | 17 | 34 | 32 | 151 | 992 | 169 | 50 | 36 | 5 | 1698 | 55 | 325 |
| **27** | 0.03 | 61 | 2423 | 9 | 51 | 71 | 98 | 506 | 99 | 472 | 37 | 26 | 3081 | 182 | 929 |
| **28** | 0.03 | 3 | 2640 | 17 | 34 | 32 | 151 | 992 | 169 | 50 | 36 | 5 | 1698 | 55 | 325 |
| **29** | 0.07 | 31 | 1751 | 17 | 42 | 72 | 87 | 669 | 104 | 370 | 82 | 21 | 2448 | 186 | 790 |
| **30** | 0.03 | 42 | 2564 | 6 | 35 | 88 | 151 | 862 | 166 | 57 | 36 | 10 | 1286 | 151 | 459 |
| **31** | 0.06 | 86 | 1401 | 18 | 31 | 85 | 83 | 539 | 88 | 194 | 115 | 27 | 1882 | 224 | 902 |
| **32** | 0.03 | 18 | 2080 | 18 | 43 | 78 | 154 | 651 | 141 | 50 | 114 | 26 | 1228 | 251 | 300 |
| **33** | 0.12 | 92 | 883 | 6 | 12 | 71 | 77 | 416 | 55 | 294 | 99 | 11 | 853 | 209 | 709 |
| **34** | 0.08 | 47 | 796 | 7 | 13 | 70 | 80 | 469 | 59 | 21 | 108 | 15 | 273 | 195 | 213 |
| **35** | 0.53 | 1000 | 3050 | 28 | 93 | 133 | 462 | 582 | 119 | 11099 | 172 | 17 | 658 | 371 | 2326 |
| **36** | 0.27 | 128 | 3175 | 9 | 58 | 129 | 122 | 554 | 98 | 894 | 219 | 8 | 638 | 341 | 708 |
| **37** | 0.09 | 94 | 4891 | 5 | 204 | 242 | 369 | 571 | 273 | 692 | 91 | 6 | 2492 | 550 | 517 |

| | | | | | | | | | | | | | | | |
|---|---|---|---|---|---|---|---|---|---|---|---|---|---|---|---|
| **38** | 0.03 | 8 | 2438 | 10 | 31 | 54 | 133 | 1781 | 135 | 98 | 3.5 | 1.5 | 1266 | 86 | 1147 |
| **39** | 0.03 | 3 | 1845 | 7 | 20 | 34 | 115 | 2079 | 136 | 89 | 3.5 | 1.5 | 964 | 47 | 408 |
| **40** | 0.03 | 10 | 1883 | 4 | 21 | 72 | 136 | 1214 | 107 | 82 | 3.5 | 8 | 1228 | 163 | 841 |
| **41** | 0.13 | 32 | 795 | 11 | 8 | 54 | 89 | 899 | 52 | 82 | 3.5 | 3 | 289 | 174 | 299 |
| **42** | 0.13 | 59 | 2693 | 5 | 34 | 97 | 240 | 768 | 158 | 91 | 3.5 | 6 | 2214 | 334 | 6243 |
| **43** | 0.19 | 34 | 1470 | 2 | 12 | 94 | 86 | 307 | 54 | 57 | 78 | 1.5 | 545 | 308 | 1139 |
| **44** | 0.07 | 50 | 1541 | 11 | 71 | 106 | 130 | 445 | 182 | 82 | 28 | 7 | 1014 | 352 | 1257 |
| **45** | 0.03 | 8 | 2940 | 14 | 37 | 46 | 144 | 1295 | 174 | 65 | 3.5 | 1.5 | 2415 | 125 | 209 |
| **46** | 0.03 | 3 | 5039 | 2 | 83 | 130 | 183 | 428 | 177 | 100 | 3.5 | 12 | 4168 | 366 | 168 |
| **47** | 0.03 | 29 | 3387 | 11 | 65 | 125 | 155 | 482 | 166 | 89 | 3.5 | 6 | 2287 | 402 | 163 |
| **48** | 0.03 | 24 | 3475 | 4 | 74 | 138 | 157 | 362 | 167 | 98 | 3.5 | 7 | 2277 | 419 | 239 |
| **49** | 0.11 | 36 | 1169 | 10 | 11 | 69 | 72 | 382 | 50 | 32 | 35 | 4 | 605 | 255 | 195 |
| **50** | 0.03 | 35 | 3787 | 2 | 29 | 47 | 134 | 1581 | 120 | 60 | 3.5 | 1.5 | 1786 | 171 | 350 |
| **51** | 0.03 | 11 | 3929 | 4 | 20 | 83 | 127 | 1431 | 124 | 59 | 3.5 | 3 | 2112 | 165 | 283 |
| **52** | 0.06 | 55 | 3326 | 5 | 51 | 130 | 117 | 616 | 152 | 75 | 16 | 8 | 2335 | 295 | 532 |
| **53** | 0.15 | 59 | 1425 | 5 | 12 | 90 | 76 | 430 | 64 | 65 | 34 | 5 | 655 | 266 | 435 |
| **54** | 0.08 | 47 | 2944 | 8 | 38 | 106 | 112 | 683 | 137 | 64 | 3.5 | 9 | 2116 | 244 | 216 |
| **55** | 0.13 | 29 | 1613 | 6 | 22 | 107 | 74 | 382 | 68 | 42 | 42 | 5 | 735 | 276 | 349 |
| **56** | 0.14 | 59 | 1153 | 6 | 5 | 81 | 65 | 332 | 46 | 26 | 53 | 4 | 449 | 244 | 354 |
| **57** | 0.04 | 3 | 1678 | 6 | 212 | 13 | 574 | 1175 | 112 | 8 | 66 | 1.5 | 1522 | 54 | 782 |
| **58** | 0.04 | 3 | 2913 | 10 | 40 | 25 | 160 | 1235 | 54 | 56 | 41 | 1.5 | 820 | 165 | 420 |
| **59** | 0.04 | 3 | 2168 | 5 | 49 | 18 | 668 | 1202 | 134 | 46 | 69 | 6 | 1631 | 80 | 419 |
| **60** | 0.04 | 13 | 2148 | 2 | 59 | 68 | 232 | 1291 | 628 | 149 | 25 | 1.5 | 1981 | 70 | 1525 |
| **61** | 0.11 | 3 | 1923 | 2 | 18 | 3 | 179 | 1300 | 56 | 108 | 16 | 1.5 | 1919 | 51 | 878 |
| **62** | 0.09 | 3 | 1806 | 2 | 17 | 3 | 172 | 1301 | 18 | 112 | 26 | 1.5 | 1803 | 48 | 625 |
| **63** | 0.1 | 3 | 2003 | 2 | 21 | 3 | 167 | 1157 | 8 | 98 | 21 | 1.5 | 1779 | 64 | 660 |
| **64** | 0.32 | 55 | 5279 | 2 | 71 | 154 | 216 | 716 | 71 | 128 | 253 | 4 | 877 | 408 | 375 |
| **65** | 0.14 | 34 | 1078 | 1 | 36 | 93 | 379 | 789 | 92 | 111 | 228 | 79 | 742 | 307 | 1214 |
| **66** | 0.1 | 20 | 3648 | 1 | 82 | 70 | 73 | 899 | 120 | 245 | 94 | 1 | 3198 | 278 | 976 |
| **67** | 0.14 | 34 | 1078 | 1 | 36 | 93 | 379 | 789 | 92 | 111 | 228 | 79 | 742 | 307 | 1214 |
| **68** | 0.06 | 38 | 2821 | 1 | 74 | 285 | 164 | 1374 | 254 | 117 | 68 | 9 | 2359 | 287 | 263 |
| **69** | 0.08 | 43 | 2301 | 1 | 48 | 262 | 148 | 1362 | 217 | 157 | 87 | 1 | 1948 | 243 | 182 |
| **70** | 0.07 | 3 | 1566 | 2 | 75 | 168 | 63 | 782 | 91 | 118 | 203 | 1.5 | 1227 | 314 | 309 |
| **71** | 0.06 | 14 | 3507 | 2 | 127 | 270 | 103 | 1224 | 329 | 223 | 103 | 1.5 | 2579 | 385 | 588 |
| **72** | 0.27 | 26 | 4397 | 2 | 78 | 221 | 90 | 1739 | 224 | 212 | 41 | 1.5 | 3727 | 222 | 289 |
| **73** | 0.08 | 46 | 4580 | 2 | 107 | 198 | 106 | 1065 | 209 | 144 | 43 | 1.5 | 3314 | 283 | 240 |
| **74** | 0.04 | 19 | 993 | 1 | 50 | 76 | 461 | 1798 | 122 | 42 | 61 | 17 | 830 | 166 | 2751 |
| **75** | 0.04 | 19 | 1348 | 1 | 53 | 70 | 318 | 1940 | 116 | 154 | 46 | 14 | 1091 | 128 | 1493 |

| | | | | | | | | | | | | | | | |
|---|---|---|---|---|---|---|---|---|---|---|---|---|---|---|---|
| **76** | 0.04 | 3 | 2580 | 2 | 65 | 34 | 208 | 1607 | 576 | 85 | 46 | 4 | 1314 | 43 | 776 |
| **77** | 0.12 | 3 | 2508 | 2 | 74 | 102 | 265 | 1960 | 92 | 175 | 57 | 5 | 1419 | 306 | 5024 |
| **78** | 0.1 | 3 | 2787 | 2 | 160 | 92 | 276 | 3121 | 189 | 161 | 44 | 1.5 | 1934 | 287 | 821 |
| **79** | 0.04 | 15 | 1378 | 1 | 98 | 60 | 419 | 1862 | 243 | 139 | 34 | 13 | 1113 | 77 | 630 |
| **80** | 0.1 | 7 | 394 | 1 | 31 | 98 | 70 | 1185 | 66 | 43 | 22 | 7 | 188 | 106 | 333 |
| **81** | 0.04 | 1 | 2932 | 1 | 61 | 3 | 97 | 1646 | 93 | 82 | 24 | 1 | 1942 | 83 | 4387 |
| **82** | 0.04 | 1 | 3129 | 1 | 74 | 3 | 158 | 1439 | 100 | 110 | 32 | 1 | 2779 | 164 | 3341 |
| **83** | 0.04 | 1 | 2793 | 1 | 83 | 3 | 135 | 1421 | 150 | 71 | 37 | 1 | 1643 | 95 | 1873 |
| **84** | 0.04 | 3 | 2206 | 2 | 54 | 67 | 2687 | 1599 | 123 | 95 | 57 | 1.5 | 982 | 126 | 4729 |
| **85** | 0.06 | 1 | 819 | 1 | 23 | 602 | 110 | 1775 | 333 | 77 | 72 | 1 | 412 | 97 | 422 |
| **86** | 0.08 | 4 | 706 | 1 | 44 | 581 | 100 | 1556 | 322 | 74 | 88 | 4 | 412 | 123 | 314 |
| **87** | 0.09 | 5 | 530 | 1 | 65 | 705 | 93 | 1339 | 391 | 54 | 81 | 1 | 346 | 110 | 380 |
| **88** | 0.06 | 1 | 513 | 1 | 71 | 601 | 89 | 1314 | 333 | 66 | 99 | 1 | 356 | 125 | 251 |
| **89** | 0.06 | 12 | 472 | 1 | 57 | 553 | 83 | 1301 | 307 | 57 | 102 | 1 | 357 | 123 | 236 |
| **90** | 0.05 | 2 | 501 | 1 | 39 | 385 | 82 | 1492 | 209 | 73 | 120 | 7 | 493 | 167 | 201 |
| **91** | 0.01 | 7 | 512 | 1 | 41 | 416 | 82 | 1476 | 227 | 62 | 126 | 3 | 488 | 179 | 222 |
| **92** | 0.04 | 1 | 413 | 1 | 68 | 420 | 75 | 1109 | 240 | 51 | 117 | 5 | 379 | 154 | 296 |
| **93** | 0.04 | 3 | 2467 | 2 | 93 | 588 | 131 | 1957 | 260 | 96 | 49 | 1.5 | 2191 | 102 | 323 |
| **94** | 0.05 | 3 | 2400 | 2 | 41 | 472 | 108 | 1315 | 224 | 25 | 28 | 1.5 | 826 | 50 | 290 |
| **95** | 0.06 | 3 | 2710 | 2 | 47 | 380 | 95 | 1366 | 249 | 6 | 9 | 1.5 | 832 | 46 | 398 |
| **96** | 0.05 | 3 | 2908 | 2 | 64 | 965 | 115 | 1420 | 303 | 9 | 14 | 1.5 | 1026 | 60 | 392 |
| **97** | 0.09 | 1 | 2717 | 1 | 10 | 63 | 35 | 918 | 5 | 111 | 167 | 1 | 526 | 239 | 1140 |
| **98** | 0.26 | 39 | 1437 | 1 | 11 | 408 | 120 | 1752 | 513 | 156 | 112 | 2 | 413 | 154 | 965 |
| **99** | 0.06 | 1 | 1775 | 1 | 85 | 215 | 293 | 1214 | 307 | 243 | 63 | 1 | 919 | 269 | 487 |
| **100** | 0.09 | 1 | 1772 | 1 | 86 | 232 | 325 | 1215 | 276 | 277 | 60 | 1 | 970 | 380 | 403 |
| **101** | 0.04 | 1 | 1918 | 1 | 70 | 196 | 246 | 1477 | 289 | 80 | 38 | 1 | 937 | 192 | 308 |
| **102** | 0.06 | 1 | 1853 | 1 | 81 | 106 | 299 | 1588 | 260 | 164 | 52 | 1 | 983 | 273 | 307 |
| **103** | 0.07 | 1 | 1626 | 1 | 65 | 223 | 190 | 1531 | 262 | 5 | 9 | 1 | 845 | 206 | 216 |
| **104** | 0.04 | 3 | 2993 | 8 | 490 | 19 | 344 | 3078 | 184 | 83 | 45 | 1.5 | 1673 | 71 | 732 |
| **105** | 0.04 | 3 | 3293 | 7 | 101 | 45 | 337 | 2681 | 259 | 227 | 79 | 1.5 | 1792 | 162 | 459 |
| **106** | 0.04 | 3 | 2555 | 2 | 81 | 10 | 335 | 3310 | 235 | 7 | 38 | 6 | 1491 | 65 | 309 |
| **107** | 0.04 | 3 | 3309 | 2 | 80 | 80 | 489 | 1706 | 1187 | 176 | 131 | 4 | 2181 | 412 | 375 |
| **108** | 0.04 | 8 | 2677 | 11 | 92 | 115 | 472 | 1751 | 362 | 180 | 114 | 1.5 | 2035 | 394 | 348 |
| **109** | 0.04 | 3 | 2590 | 2 | 82 | 104 | 455 | 1782 | 395 | 209 | 129 | 5 | 2059 | 381 | 335 |
| **110** | 0.04 | 3 | 1962 | 5 | 44 | 161 | 293 | 1064 | 209 | 228 | 179 | 5 | 888 | 387 | 393 |
| **111** | 0.04 | 3 | 1662 | 2 | 38 | 166 | 230 | 929 | 154 | 224 | 198 | 1.5 | 603 | 362 | 368 |
| **112** | 0.25 | 18 | 4295 | 1 | 72 | 113 | 364 | 808 | 181 | 146 | 97 | 13 | 3922 | 463 | 221 |
| **113** | 0.1 | 19 | 2328 | 2 | 41 | 146 | 1709 | 1158 | 153 | 205 | 78 | 1.5 | 2293 | 478 | 1092 |

| | | | | | | | | | | | | | | | |
|---|---|---|---|---|---|---|---|---|---|---|---|---|---|---|---|
| **114** | 0.03 | 9 | 1471 | 2 | 33 | 63 | 133 | 1329 | 67 | 45 | 14 | 1.5 | 547 | 137 | 654 |
| **115** | 0.03 | 23 | 1080 | 2 | 43 | 84 | 126 | 1197 | 95 | 55 | 41 | 1.5 | 346 | 227 | 649 |
| **116** | 0.03 | 31 | 1436 | 2 | 31 | 68 | 111 | 1017 | 44 | 45 | 33 | 1.5 | 518 | 169 | 459 |
| **117** | 0.03 | 6 | 3527 | 2 | 55 | 117 | 236 | 1992 | 152 | 111 | 87 | 1.5 | 2025 | 277 | 1735 |
| **118** | 0.03 | 6 | 5437 | 17 | 50 | 67 | 229 | 1627 | 92 | 48 | 83 | 1.5 | 2470 | 149 | 6793 |
| **119** | 0.03 | 6 | 2810 | 2 | 62 | 62 | 199 | 1627 | 86 | 10 | 48 | 1.5 | 1268 | 134 | 2202 |
| **120** | 0.03 | 17 | 3160 | 1 | 37 | 558 | 366 | 2837 | 492 | 163 | 115 | 3 | 901 | 424 | 351 |
| **121** | 0.04 | 15 | 2636 | 2 | 92 | 435 | 406 | 2376 | 410 | 184 | 106 | 4 | 880 | 578 | 355 |
| **122** | 0.03 | 21 | 5843 | 1 | 1.5 | 633 | 310 | 5231 | 411 | 102 | 39 | 1 | 1130 | 252 | 270 |
| **123** | 0.03 | 22 | 3301 | 1 | 1.5 | 518 | 314 | 3753 | 372 | 139 | 95 | 1 | 858 | 342 | 261 |
| **124** | 0.07 | 12 | 3012 | 1 | 20 | 376 | 250 | 904 | 365 | 184 | 92 | 3 | 3213 | 249 | 537 |
| **125** | 0.07 | 7 | 2167 | 3 | 1.5 | 412 | 221 | 826 | 287 | 100 | 82 | 3 | 2683 | 258 | 277 |
| **126** | 0.25 | 101 | 3208 | 2 | 53 | 609 | 165 | 1757 | 464 | 353 | 33 | 34 | 1741 | 295 | 2073 |
| **127** | 0.08 | 16 | 3634 | 2 | 113 | 362 | 196 | 2542 | 428 | 156 | 39 | 1.5 | 2594 | 371 | 276 |
| **128** | 0.06 | 3 | 2845 | 2 | 91 | 440 | 243 | 1687 | 311 | 121 | 73 | 1.5 | 2311 | 199 | 262 |
| **129** | 0.09 | 3 | 2862 | 2 | 54 | 287 | 278 | 988 | 278 | 174 | 117 | 1.5 | 2964 | 312 | 341 |
| **130** | 0.05 | 3 | 1666 | 2 | 71 | 356 | 223 | 687 | 344 | 151 | 125 | 1.5 | 3399 | 393 | 398 |
| **131** | 0.02 | 7 | 3512 | 1 | 112 | 431 | 404 | 1226 | 229 | 202 | 51 | 1 | 1712 | 378 | 422 |
| **132** | 0.005 | 3 | 3484 | 2 | 9 | 220 | 281 | 1351 | 202 | 147 | 52 | 1.5 | 1410 | 139 | 269 |
